# Prognosis tools for short-term adverse events in older emergency department users: result of a Québec observational prospective cohort

**DOI:** 10.1186/s12877-020-01999-6

**Published:** 2021-01-22

**Authors:** Cyrille P. Launay, Joshua Lubov, Kevin Galery, Christine Vilcocq, Éric Maubert, Marc Afilalo, Olivier Beauchet

**Affiliations:** 1 Department of Medicine, Division of Geriatric Medicine, Sir Mortimer B. Davis - Jewish General Hospital and Lady Davis Institute for Medical Research, McGill University, 3755 chemin de la Côte Sainte-Catherine, Montreal, Quebec H3T 1E2 Canada; 2 Centre of Excellence on Longevity of McGill Integrated University Health and Social services Network, Quebec, Canada; 3 Emergency Department, Jewish General Hospital, McGill University, Montreal, Quebec Canada; 4 Dr. Joseph Kaufmann Chair in Geriatric Medicine, Faculty of Medicine, McGill University, Montreal, Quebec Canada; 5 Lee Kong Chian School of Medicine, Nanyang Technological University, Singapore, Indonesia

**Keywords:** Emergency department, Adverse event, Prognosis, Older adults

## Abstract

**Background:**

The “Program of Research on the Integration of Services for the Maintenance of Autonomy” (PRISMA-7) and “Emergency room evaluation and recommendations” (ER^2^) are both clinical tools used in Québec Emergency Departments (EDs) for screening of older ED users at higher risk of poor outcomes, such as prolonged length of stay (LOS) in EDs and in hospital. The study aimed to: 1) examine whether the PRISMA-7 and ER^2^ risk levels were associated with length of stays in ED and hospital, as well as hospital admission; and 2) compare the criteria performance (i.e.*,* sensitivity, specificity, positive predictive value, negative predictive value, likelihood ratios and area under receiver operating characteristic curve) of the PRISMA-7 and ER2 high-risk levels for these three ED adverse events in Québec older patients visiting ED on a stretcher.

**Methods:**

A total of 1905 older patients who visited the ED of the Jewish General Hospital (Montreal, Québec, Canada) on stretchers were recruited in this prospective observational cohort. Upon their ED arrival, PRISMA-7 and ER^2^ were performed. The outcomes were LOS in ED and in hospital, and hospital admission.

**Results:**

The PRISMA-7 and ER^2^ risk levels were associated with length of stay in ED and hospital as well as with hospital admission. Prolonged stays and higher hospitalization rates were associated with high-risk levels, whereas those in low-risk level groups had significantly shorter LOS and a lower rate of hospital admission (*P* < 0.006). While performance measures were poor for both assessment tools, ER^2^ had a greater prognostic testing accuracy compared with PRISMA-7.

**Conclusion:**

PRISMA-7 and ER^2^ were both associated with incidental short-term ED adverse events but their overall prognostic testing accuracy was low, suggesting that they cannot be used as prognostic tools for this purpose.

## Background

Overcrowding and delays in providing care have become increasingly problematic in emergency departments (EDs) in high-income countries [1, 2]. The limited access to primary care in Canada, in part related to the lack of primary care physicians, force people in the community to seek care in EDs ([1, 2]. A continuously enlarging proportion of ED users are aged over 75 and account for up to a quarter of all ED users. These ED users, compared to their younger counterparts, are at higher risk for short-term ED adverse events including prolonged length of stay in ED and in hospital, as well as hospital admission during an index ED visit ([2], http://www.csbe.gouv.qc.ca/fileadmin/www/2016/Urgences/CSBE_Rapport_Urgences_2016.pdf). A major concern of ED healthcare workers is the identification of older patients who are the most vulnerable and are at highest risk of adverse outcomes (e.g. long ED and hospital stay) and treating them in a timely manner [1–4]. The occurrence of adverse events may be reduced if the older ED users’ risk is determined early in the process of ED care (i.e.*,* upon their ED arrival) [1, 3, 4]. One way for preventing short-term ED adverse events is to screen older users with the highest risk and provide timely and individualized geriatric interventions [5–9]. Therefore, the prognostic tools designed for this purpose may provide relevant information regarding potential adverse events early in the ED care plan, with the goal of improving the decision-making process and to reduce adverse events [10–15]. The identification of older patients at risk for short-term ED adverse events is, therefore, an important objective with regards to optimizing the care provided in the EDs. The “Program of Research on the Integration of Services for the Maintenance of Autonomy” (PRISMA-7) is a tool used to assess older ED users in the province of Québec (Canada) [16, 17]. PRISMA-7 was designed to screen disabilities in older community-dwellers and it stratifies them into two risk levels: low versus high-risk for disabilities. Disabilities are the result of chronic diseases burden, among which musculoskeletal and cardiovascular diseases are the main contributors [18]. Regardless the reasons of the ED visit, disabilities and related burden of chronic diseases characterize older ED users and this poorer overall health status may explain in part the high prevalence of the adverse events, suggesting that PRISMA-7 may be used as a prognosis tool to predict short-term ED adverse events [1–4, 16, 17]. To date, the association of the PRISMA-7 high-risk level with the occurrence of short-term ED adverse events has never been examined and therefore validated as a prognosis tool for this purpose. *“Emergency room evaluation and recommendations”* (ER^2^) is another clinical tool currently used in Québec EDs [4, 19]. Compared to PRISMA-7, ER^2^ has been particularly designed for assessing the risk for short-term ED adverse events in older patients [12–14]. The objective of the present study is to determine which clinical tool, between PRISMA-7 and ER^2^ is the most appropriate tool to use in EDs for detecting the older ED users most at risk for short-term ED adverse events. No previous study has compared PRISMA-7 and ER^2^ high-risk levels with regards to predicting the occurrence of the short-term ED adverse events. Firstly, we hypothesised that both PRISMA-7 and ER^2^ high-risk levels would be associated with the occurrence of short-term ED adverse events. Disabilities of older ED users and related chronic diseases burden may explain in part the occurrence of the short-term ED adverse events [1–4]. PRISMA-7 screens disabilities, suggesting that it may be used as a prognostic tool of these ED adverse events [16, 17]. Secondly, we hypothesized that ER^2^ would be a significantly better prognostic tool compared to PRISMA-7 for predicting prolonged length of stay in ED and in hospital, as well as predicting hospital admission, because it was designed and validated for this specific goal unlike PRISMA-7 [4, 12–14, 19].

The study aimed to: 1) examine whether the PRISMA-7 and ER^2^ high-risk levels were associated with length of stays in ED and in hospital, as well as hospital admission, an 2) compare the performance criteria (i.e.*,* sensitivity, specificity, positive predictive value [PPV], negative predictive value [NPV], likelihood ratios [LR] and area under receiver operating characteristic curve) of the PRISMA-7 and ER2 high-risk levels for the three previously stated ED adverse events in Québec older patients visiting ED on a stretcher.

## Methods

### Population and design

Older adults who consulted the ED of the Jewish General Hospital (Montreal, Quebec, Canada) between September 2017 and January 2018 were potential participants in this prospective observational cohort study. The inclusion criteria of the present study were: age ≥ 75, unplanned ED visit, being on a stretcher (non-ambulatory), assessed with PRISMA-7 and ER^2^ and agree to participate in the study. The exclusion criteria were participation in an experimental study, being in palliative care and transfer to another hospital during the ED visit. A total of 7017 older users aged ≥75 visited the ED of the Jewish General Hospital during the period of recruitment and 1905 (27.2%) among this group satisfied all selection criteria.

### Baseline assessment

Firstly, PRISMA-7 was completed by the nurse in charge of the triage for medico-surgical emergencies. The nurse asked patients and/or their family the PRISMA-7 questions, details of PRISMA-7 items have been previously described [17]. The scoring method of PRISMA-7 is to assign 1 point to the answer *yes* and no points to the answer *no,* and to stratify patients into two risk levels: low (i.e.*,* score 0 to 2) and high (i.e.*,* score 3 to 7) risk [17].

Secondly, once the patient is on a stretcher and the triage is completed, their nurse completed the ER^2^ assessment. ER^2^ is composed of 6 close-ended format questions (i.e., yes versus no) that have been described previously in detail [4, 19]. Compared to PRISMA-7, which has a uniform scoring system, the ER^2^ is divided into major and minor criteria with different assigned scores. A score of five points is assigned to each major criteria, which include “use of walking aid” and “temporal disorientation”, whereas for the minor criteria, which comprise the other four criteria of ER^2^ a score of 1 point is assigned. The range of score is 0 (lowest risk) to 14 (highest risk). ER^2^ stratifies risk for short-term ED adverse events into three levels based on obtained score: low (0-3), moderate [3, 4] and high (≥ 6).

In addition, the following items were recorded using the patients’ electronic medical record: age, sex, and place of living (home versus residence versus other). The reasons for ED visits were categorized into 5 sub-types: organ failure defined as an acute organ decompensation; mobility disorders defined as gait and/or balance impairment and/or fall with or without fall-related injuries; neuropsychiatric disorders defined as delirium, dementia, and/or behavioral disorders; cancer defined as a group of diseases involving abnormal cell growth with the potential to invade or spread to other parts of the body; and social issues defined as the absence of acute disease symptoms combined with an acute increase in the use of formal and/or informal home and social services leading to an inability to cope at home.

This study has been performed following the usual procedure of the ED of the Jewish General Hospital. PRISMA-7 in this hospital is usually performed at initial triage, which is the first step of assessment of ED users. The triage is a process with the purpose of prioritizing patients depending on the nature and urgency of their health condition and to identify those require urgent evaluation and treatment. This first step of ED visit assesses the type and severity of patient health conditions, determines access to appropriate treatments and assigns appropriate human health resources. The Canadian ED Triage and Acuity Scale was used in this study [20]. This scale is composed of 5 levels of urgencies which are: level 1, defined as resuscitation; level 2, defined as emergent; level 3, defined as urgent; level 4, defined as less urgent; and level 5, defined as non-urgent. The triage process thus determines whether the ED users need to be seen as ambulatory patients (level 4 and 5) or on a stretcher (level 2 and 3). There is no need to perform ER^2^ in ambulatory patient, because this category of ED users is habitually discharged home after their ED visit. Since ER^2^ is a tool to screen older ED users at risk for short-term ED adverse events, it has only been performed in older ED users with level 2 and 3 who were on stretchers (non-ambulatory). PRISMA-7 and ER^2^ assessments were therefore performed at different times, but the delay between the two assessments was short (less than 1 h) for older ED users on stretcher.

### Follow-up and outcomes

The dates and hours of ED visit, hospital admission and discharge were extracted from the patients’ digital file when the patient was discharged from ED or hospital. The length of stay in ED, expressed in hours, was defined as the delay between the hour and day of the ED visit and the hour and day of the ED discharge (either to hospital or place of living). This outcome was used as the primary outcome. The length of stay in hospital, expressed in days, was defined as the delay between day of the ED visit and the day of the hospital discharge. The highest tertile of length of stay in ED (i.e., > 26.9 h) and hospital (i.e., > 13 days) defined a prolonged length of stay. Hospital admission after an index ED visit was considered when older ED users were admitted to medical or surgical wards at the Jewish General Hospital.

### Power analysis

The number of participants required for this study has been calculated to have enough statistical power in order to compare the high-risk group to the low-risk group for the primary outcome, ED length of stay, as well as for the secondary outcomes (length of stay in hospital and hospital admission). Based on a pilot study, comparisons to be made between high-risk and low-risk groups are: the length of stay in ED (23.4 ± 13.5 h versus 18.9 ± 13.1 h), the length of stay in hospital (21.8 ± 17.3 days versus 15.8 ± 14.5 days), and hospital admission (58.6% versus 40.2%). In order to detect differences for these 3 outcomes with power 90% at the 0.05 significance level, this study needed 1316 participants for ED length of stay, 1896 for hospital length of stay and 1901 for hospital admission.

### Ethics

This study was performed in accordance with the ethical standards set forth in the Helsinki Declaration (1983). All participants recruited in this study provided a verbal informed consent because the study did not change the usual clinical practice. The verbal informed consent was obtained from the patients themselves in the presence of their trusted person, usually a family member, who helped them to make decision. Participants or their legal guardian when appropriate, were informed that their medical information may be used for research purpose. If they disagreed, they informed the physician taking care of them and a note was recorded in their chart. The Research Review Office of the Jewish General Hospital and the Research Ethic Committee of the Integrated Health and Social Services University Network for West-Central Montreal approved this process.

### Statistics

Means and standard deviations (SD) or frequencies were used to characterize the participants’ baseline characteristics. Between group-comparisons were performed using analysis of variance (ANOVA) with Bonferroni correction for multiple comparisons, unpaired *t*-test and Chi-square, as appropriate. Multiple linear and logistic regressions examined the association of the PRISMA-7 and ER^2^ risk levels (used as independent variable with separated model for each tool and risk levels) with length of stay in ED and in hospital, and hospital admission (used as dependent variable with separated model for each variable) adjusted by place of living and reasons for ED visit. The performance criteria examined were sensitivity, specificity, positive predictive value, negative predictive value, likelihood ratios and area under receiver operating characteristic curve. The elapsed time to discharge from ED and hospital for PRISMA-7 and ER^2^ risk levels was examined by survival Kaplan-Meier curves and log-rank test. *P*-values < 0.05 were considered statistically significant. All statistics were performed using SPSS (version 24.0; SPSS, Inc., Chicago, IL).

## Results

Table 1 shows the comparison of baseline patient’s characteristics separated into different subgroups based on their PRISMA-7 (i.e.*,* low versus high) and ER^2^ risk levels (i.e.*,* low, moderate and high). Patients with a PRISMA-7 high-risk level compared to those with low-risk level were older (*P* ≤ 0.001) and more frequently male (*P* ≤ 0.001); lived more often at home (*P* = 0.001) with support (*P* = 0.009), and had more frequently polypharmacy (*P* ≤ 0.001), temporal disorientation (*P* = 0.001) and walking aid (*P* ≤ 0.001). They had also a longer length of stay in ED (*P* = 0.002) and in hospital (*P* = 0.004), and were more frequently admitted to hospital (*P* = 0.003) compared to those with a PRISMA-7 low-risk level. Similar results for age, sex, home support, polypharmacy, temporal disorientation and use of walking aid were observed for comparisons between the three ER^2^ risk levels. More serious presenting conditions and higher incidence of adverse events were found in high-risk levels compared to moderate-risk and low-risk levels, as well as between moderate-risk and low-risk levels (*P* ≤ 0.003). There was no significant different for place of living (*P* = 0.784) between mild and moderate-risk levels, as well as no significant difference for sex (*P* = 0.453) between mild and high-risk levels, and no significant difference for home support (*P* = 0.651) and polypharmacy (*P* = 0.430) between moderate and high-risk levels. Furthermore, neuropsychiatric disorders as a reason for ED visit was more prevalent in patients with low-risk levels compared to those in moderate-risk group (*P* = 0.022), but less prevalent compared to high-risk group (*P* = 0.036). Prevalence of this reason of ED visit was also higher in high-risk group of patients compared to those in moderate-risk group (*P* = 0.005). Organ failure was also a more prevalent reason of ED visit in patients with a moderate compared to a high-risk level (*P* = 0.025). In addition, longer stay in ED and hospital as well as hospital admission were observed in patients considered to have high-risk level compared to those with low-risk level (*P* ≤ 0.001). Hospital admission was also more prevalent in patients with the moderate-risk level compared to those with the low-risk level (*P* = 0.040).

**Table 1:** Comparison of baseline characteristics of participants on stretcher visiting emergency department separated in different subgroups based on their PRISMA-7 and ER^2^ scores (*n* = 1905)

	PRISMA-7 risk			ER^2^ risk			*P*-value^d^		
	Low^a^ (*n* = 885)	High^b^ (*n* = 1020)	*P*-value^c^	Low (*n* = 722)	Moderate (*n* = 70)	High (*n* = 1113)	Low versus moderate	Low versus high	Moderate versus high
Age (years)									
Mean ± SD	84.5 ± 6.0	86.1 ± 6.0	**≤0.001**	83.2 ± 5.4	86.5 ± 5.2	86.7 ± 6.1	**≤0.001**	**≤0.001**	1.000
≥ 85 years	406 (45.9)	584 (57.3)	**≤0.001**	250 (34.6)	55 (78.6)	685 (61.5)	**≤0.001**	**≤0.001**	**0.004**
Male	311 (35.1)	497 (48.7)	**≤0.001**	304 (42.1)	55 (78.6)	449 (40.3)	**≤0.001**	0.453	**≤0.001**
Place of living home	553 (62.5)	714 (70.0)	**0.001**	547 (75.8)	52 (74.3)	668 (60.0)	0.784	**≤0.001**	**0.018**
Home support	565 (63.8)	709 (69.5)	**0.009**	320 (44.3)	55 (78.6)	899 (80.8)	**≤0.001**	**≤0.001**	0.651
Polypharmacy^e^	600 (67.8)	806 (79.0)	**≤0.001**	435 (60.2)	55 (78.6)	916 (82.3)	**0.003**	**≤0.001**	0.430
Temporal disorientation^f^	196 (22.1)	294 (28.8)	**0.001**	0	3 (4.3)	487 (43.8)	**≤0.001**	**≤0.001**	**≤0.001**
Use of walking aid	353 (39.9)	592 (58.0)	**≤0.001**	0	12 (17.1)	933 (83.8)	**≤0.001**	**≤0.001**	**≤0.001**
Reason for ED visit, n (%)									
Organ failure^g^	508 (57.4)	544 (53.3)	0.075	411 (56.9)	47 (67.1)	594 (53.4)	0.098	0.135	**0.025**
Mobility disorders^h^	136 (15.4)	166 (16.3)	0.589	104 (14.4)	13 (18.6)	185 (16.6)	0.348	0.203	0.672
Neuropsychiatric disorders^i^	98 (11.1)	114 (11.2)	0.943	69 (9.6)	1 (1.4)	142 (12.8)	**0.022**	**0.036**	**0.005**
Cancer	24 (2.7)	36 (3.5)	0.308	24 (3.3)	3 (4.3)	33 (3.0)	0.672	0.665	0.533
Social issue^j^	119 (13.4)	160 (15.7)	0.168	114 (15.8)	6 (8.6)	159 (14.3)	0.108	0.377	0.181
Length of stay, mean ± SD									
ED (hour)	21.5 ± 13.8	23.5 ± 13.7	**0.002**	19.9 ± 13.3	23.7 ± 15.7	24.3 ± 13.7	0.078	**≤0.001**	1.000
Hospital (days)	12.4 ± 15.4	15.8 ± 20.0	**0.004**	10.8 ± 13.5	11.5 ± 18.7	16.3 ± 19.9	1.000	**≤0.001**	0.334
Hospital admission, n (%)	415 (46.9)	548 (53.7)	**0.003**	310 (42.9)	39 (55.7)	614 (55.2)	**0.040**	**≤0.001**	0.929

PRISMA-7: “Program of Research on the Integration of Services for the Maintenance of Autonomy”; ER^2^: Emergency room evaluation and recommendation; ED: Emergency department; SD: Standard deviation; ^a^Score < 3; ^b^Score ≥ 3; ^c^Comparison based on unpaired t-test; ^d^Comparison based on an analysis of variance (ANOVA) with Bonferroni correction for multiple comparisons; ^e^Number of medications daily taken ≥5; ^f^Inability to give the current year and/or months; ^g^Defined as an acute organ decompensation; ^h^Defined as gait and/or balance impairment and/or fall with or without fall-related injuries; ^i^Defined as delirium, dementia, behavioral disorders; ^j^Defined as the absence of symptoms of acute disease combined with an acute increase of the use of formal and/or informal home and social services leading to an inability to cope at home; *P*-values significant (i.e., < 0.05) in bold

Regardless of the regression model used, the PRISMA-7 high-risk level was associated with a prolonged length of ED (P ≤ 0.001) and hospital stay (*P* ≤ 0.007) (Table 2). The PRISMA-7 low-risk level was associated with a short length of stay in ED and in hospital for linear regression model (*P* ≤ 0.007). The ER^2^ high-risk level was associated with a prolonged length of ED and hospital stay, as well as elevated hospital admission rates (*P* ≤ 0.001) for all regression models. The ER^2^ low-risk level was associated with short length of stay in ED and in hospital, as well as a lower hospital admission rate (*P* ≤ 0.001).

**Table 2:** Linear and logistic regression models showing the association of the length of stay in emergency department and hospital as well as the hospital admission (dependent variable with separated model for each variable) with PRISMA-7 and ER^2^ risk (independent variable) adjusted on place of living and reasons for emergency department visit (*n* = 1905)

Dependent variable	Independent variable		Linear Regression^a, b^			Logistic Regression^a, b, c^		
			β	[95%CI]	*P*-Value	OR	[95%CI]	*P*-Value
Length of stay in emergency Department in hours	PRISMA-7 risk	High	2.28	[1.04;3.51]	**≤0.001**	1.57	[1.29;1.91]	**≤0.001**
		Low	−2.28	[−3.51;-1.04.]	**≤0.001**	0.64	[0.52;0.77]	**≤0.001**
	ER^2^ risk	Low	−4.11	-[−5.38;-2.83]	**≤0.001**	0.58	[0.47;0.72]	**≤0.001**
		Moderate	1.09	[−2.19;4.38]	0.514	0.97	[0.58;1.62]	0.903
		High	3.84	[2.58;5.09]	**≤0.001**	1.69	[1.38;2.07]	**≤0.001**
Length of stay in Hospital in days	PRISMA-7 score	Abnormal	3.21	[0.89;5.54]	**0.007**	1.48	[1.12;1.96]	**0.006**
		Normal	−3.21	[−5.54;-0.89]	**0.007**	0.68	[0.51;0.90]	**0.006**
	ER^2^ risk level	Mild	−5.17	[−7.63;-2.72]	**≤0.001**	0.49	[0.36;0.68]	**≤0.001**
		Moderate	−2.31	[−8.14;3.52]	0.438	0.62	[0.29;1.33]	0.218
		High	5.31	[2.92;7.70]	**≤0.001**	2.10	[1.55;2.85]	**≤0.001**
Hospital admission	PRISMA-7 score	Abnormal		–		1.32	[1.01;1.58]	**0.003**
		Normal				0.76	[0.63;0.91]	**0.003**
	ER^2^ risk level	Mild		–		0.59	[0.48;0.71]	**≤0.001**
		Moderate		–		1.17	[0.72;1.91]	0.516
		High		–		1.64	[1.36;1.98]	**≤0.001**

PRISMA-7: “Program of Research on the Integration of Services for the Maintenance of Autonomy”; ER^2^: Emergency Room evaluation and recommendation; β: Coefficient of regression beta; OR: Odd ratio; HR: Hazard ratio; CI: Confident interval; ^a^Model adjusted on place of living and reasons for emergency department visit; ^b^Separated model for each risk level; ^c^To be in the highest tertile with a length of stay in emergency Department > 26.9 h and in hospital > 13 days; P-values significant (i.e.*,* < 0.05) in bold

**Fig. 1:**
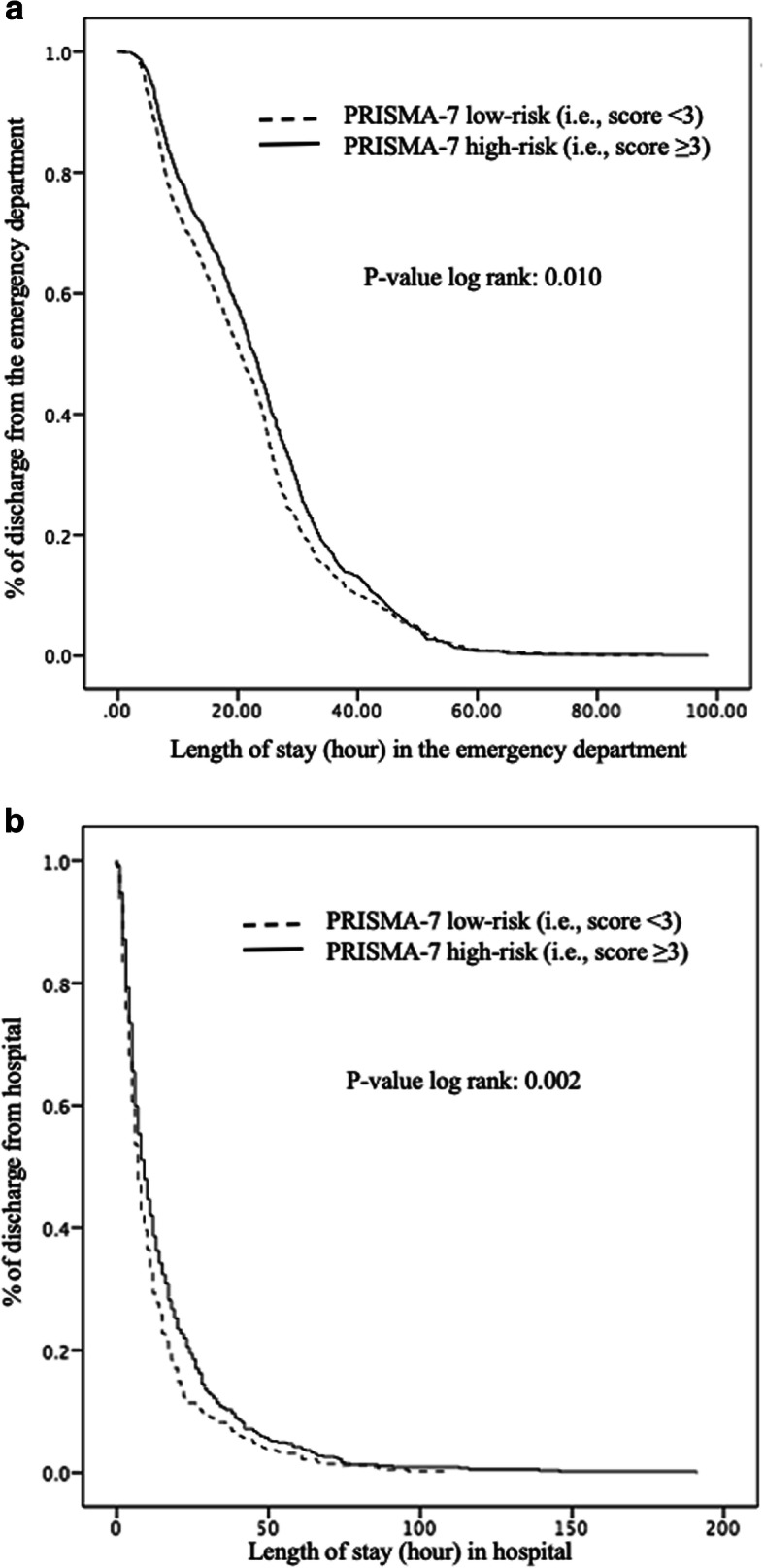
Kaplan-Meier estimates of the probability of long emergency department (**a**) and hospital (**b**) stay with PRISMA-7 risk level in participants (*n* = 1905). **a** Emergency department. **b** Hospital

**Fig. 2:**
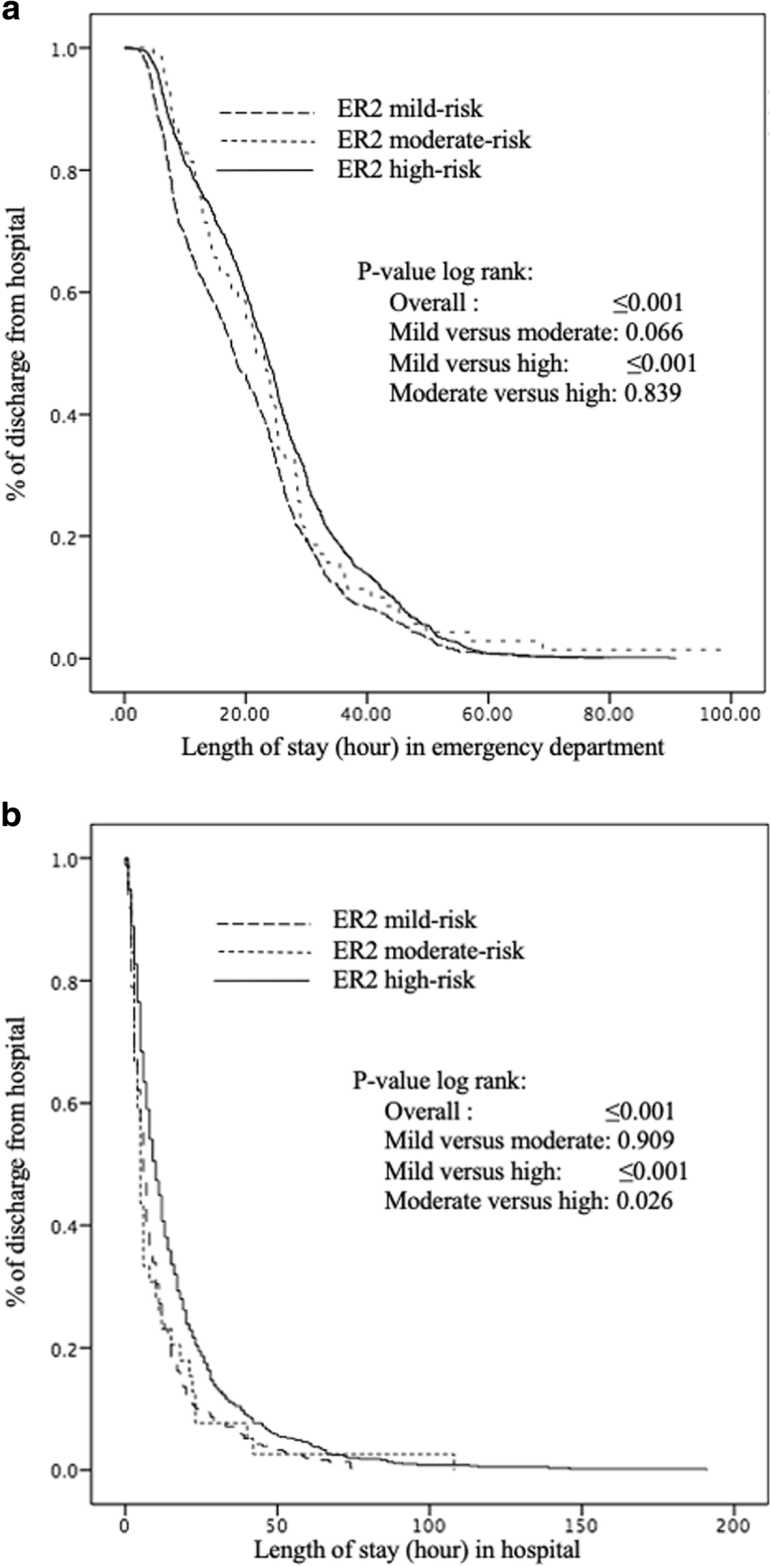
Kaplan-Meier estimates of the probability of long emergency department (**a**) and hospital (**b**) stay with ER^2^ risk levels in participants (*n* = 1905). **a** Emergency department. **b** Hospital

Kaplan-Meier distributions showed significant differences in the time elapsed before hospital discharge between patients with a PRISMA-7 low and high-risk level; those with a high-risk level were discharged from ED (*P* = 0.010) and from hospital (*P* = 0.002) later compared to those with low-risk (Fig. 1). The three ER^2^ participants’s risk levels differed significantly for length of stay in ED and in hospital (*P* ≤ 0.001), those with a high-risk level being discharged later than those in the low-risk (*P* ≤ 0.001) (Fig. 2). Furthermore, patients with a high-risk level were discharged later when hospitalized than those in the moderate-risk level (*P* = 0.026).

All performance criteria were poor (i.e.*,* sensitivity, specificity, PPV and NPV ≤0.74 LR ≤ 1.40 and area under receiver operating characteristic curve ≤0.62) with worse performances for PRISMA-7 compared to ER^2^ (Table 3).

**Table 3:** Performance criteria of PRISMA-7 and ER^2^ high-risk for long length of stay in emergency department and hospital, and hospital admission (*n* = 1905)

Criteria	Abnormal PRISMA-7 score^a^			ER^2^ high-risk level^b^		
	Length of stay		Hospital admission	Length of stay		Hospital admission
	Emergency department^c^	Hospital^d^		Emergency department^a^	Hospital^b^	
Sensitivity	0.60	0.63	0.57	0.67	0.64	0.67
Specificity	0.50	0.56	0.50	0.46	0.46	0.46
Positive predictive value	0.38	0.36	0.54	0.38	0.38	0.38
Negative predictive value	0.71	0.72	0.53	0.73	0.74	0.74
Likelihood ratio of positive test	1.20	1.18	1.14	1.25	1.25	1.25
Likelihood ratio of negative test	1.25	1.26	1.16	1.40	1.40	1.40
Area under receiver operating characteristic curve	0.55	0.56	0.53	0.59	0.62	0.56

PRISMA-7: “Program of Research on the Integration of Services for the Maintenance of Autonomy”; ER^2^: Emergency room evaluation and recommendations; ^a^Score ≥ 3/7; ^b^Score ≥ 6/14; ^c^Length of stay used as a discontinuous variable defined as the highest tertile of lengths of stay (i.e., > 26.9 h); ^d^Length of stay used as a discontinuous variable defined as the highest tertile of lengths of stay (i.e.*,* > 13 days)

## Discussion

The findings showed that PRISMA-7 and ER^2^ risk stratifications were successfully associated with length of stay in ED and in hospital, as well as hospital admission rates. High-risk levels were associated with longer stays and higher rates of hospital admission, whereas the contrary association was found in low-risk levels for both tools. The strength of association was stronger for ER^2^ compared to PRISMA-7. However, performance criteria for the high-risk level of these ED adverse events were low, with the lowest performance being reported with PRISMA-7.

PRISMA-7 and ER^2^ high-risk levels were both associated with prolonged ED and hospital stays and higher rates of hospital admission. This association was expected for ER^2^ because this clinical tool has especially been designed to determine prognosis for these short-term ED adverse events [4, 12–14, 19]. In particular, prolonged ED and hospital stays have previously been reported with the ER^2^ high-risk levels, similar to the findings in the present study [4, 12, 14, 19]. In contrast, to the best of our knowledge, it is the first time that an association with prolonged ED and hospital stays is found with the PRISMA-7 high-risk level. PRISMA-7’s initial objective was to screen disability in community dwelling older adults [16, 17]. This tool has also been used to identify frailty in older ED users, but not related adverse events [21]. Frailty may be defined as a state of vulnerability related to homeostasis following a stressor and is independently associated with adverse events [22]. Frailty results from an accumulation of severe comorbid conditions and physiological aging [22, 23]. A tool which assesses frailty and its severity indirectly evaluates prognosis for frailty-related adverse events. The main characteristic of older ED users compared to younger users is an accumulation of severe and chronic morbidities and related-disabilities  [3]. These particular health and functional conditions greatly influence the ED care plan [2, 3, 8]. Although older adults undergo more diagnostic tests and procedures than younger ED patients, their ED diagnoses tend to be less accurate [2, 7]. This has been attributed to atypical disease presentation due to multiple comorbidities, which can complicate the clinical presentation, diagnosis and care plan [1, 3, 5, 7]. Additionally, chronic morbidities may decompensate in a cascade and lead to disabilities and complications, regardless of the nature and the severity of the acute condition leading to the ED visit  [3, 22]. This statement explains why the PRISMA-7 could be used as a prognostic tool to predict prolonged ED and hospital stays. Our findings also underscore that PRISMA-7 and ER^2^ low-risk levels were associated with shorter ED and hospital stays. This finding is consistent with the former because it suggests that both tools also detect older ER users with a low level of frailty. The early identification of this category of older ED users may be useful for the ED care continuum, because these ED users with low levels of frailty have a higher propensity to be discharged to their place of living compared to those with higher stages of frailty [5–9, 24].

Our study revealed that, even if significant associations of PRISMA-7 and ER^2^ risk levels with short-term ED adverse events were found, the predictive performance was poor for both tools. No study has assessed the ability of PRISMA-7 or ER^2^ of predicting short-term ED adverse events, therefore, the comparison of this result with previous studies is difficult. However, our findings demonstrated similar results to other screening tools, such as The Identification of Seniors at Risk (ISAR), the Triage Risk Screening Tool (TRST), the eight-item questionnaire of Runciman, and the seven-item questionnaire of Rowland, if we take into consideration the ability of predicting return visits of older patients after they have been discharged from the ED. [25] Poor predictive performances were also reported in this study. The imbalance between a significant association of the PRISMA-7 and ER^2^ high-risk levels with short-term ED adverse events and the poor performance criteria highlight that a significant statistical association between a high-risk level and an adverse event does not necessarily guarantee that the prognostic tool is accurate nor reliable. Similar results have been reported with prolonged hospital stays in older patients admitted to geriatric wards [4]. Despite the limited predictive performance, improvement in cost-effectiveness of targeted geriatric interventions using a screening tool has been previously demonstrated with low positive predictive value (i.e., between 20 and 30%) [24]. In addition, screening tools are known to be particularly useful in decision-making procedures to prevent events that depend on multiple factors [25, 26]. This result confirms that screening older patients with the highest risk level and providing them a timely and appropriate intervention is an efficient strategy.

The important number of older ED users recruited and the appropriate design to achieve the research objectives are the strengths of our study. However, some limitations need to be underscored. Firstly, the recruitment has been performed in a single centre, which limits the external validation of our results. Secondly, although adjustment for clinical characteristics influencing the studied short-term adverse events was performed, there are still potential residual confounders that may influence the short-term ED adverse events. Thirdly, both PRISMA-7 and ER^2^ do not take into consideration the reasons of ED visits which is usually an acute disease directly influencing the occurrence of short-term ED adverse events. For instance, an acute disease may decompensate in a cascade related to chronic morbidities cumulated in older ED users, leading to multiple acute organ decompensations and disabilities and exposing this group to prolonged length of stays and hospitalisations [5–9]. However, in the regression analysis used to examine the association of between PRISMA-7 and ER^2^ risk levels with the three short-terms ED adverse events, all models were adjusted by the reasons of ED visits and a significant association was found.

## Conclusion

In conclusion, even if PRISMA-7 and ER^2^ high-risk levels were associated with incidental short-term ED adverse events, their performance criteria for these risks were relatively low, suggesting that they cannot be used as prognostic tools for this purpose. However, they may be the starting point of an assessment that could provide pertinent information pertaining to ED tailor-made geriatric interventions.

## Data Availability

Data and materials are available on request. Request should be sent to the corresponding author: Cyrille Launay, MD, PhD; Department of Medicine, Division of Geriatric Medicine, Sir Mortimer B. Davis - Jewish General Hospital, McGill University, 3755 chemin de la Côte-Sainte-Catherine, Montréal, QC H3T 1E2, Canada; E-mail: . All requests needs a cover letter explaining the objective, justification and the referent ethic committee.

## Abbreviations

PRISMA-7: Program of Research on the Integration of Services for the Maintenance of Autonomy
ER^2^: Emergency room evaluation and recommendations
EDs: Emergency departments
SD: Standard deviations
ANOVA: Analysis of variance
ISAR: Identification of Seniors at Risk
TRST: Triage Risk Screening Tool

## References

[1] Carpenter CR, Heard K, Wilber S, Ginde AA, Stiffler K, Gerson LW (2011). Society for Academic Emergency Medicine (SAEM) geriatric task force. Research priorities for high-quality geriatric emergency care: medication management, screening, and prevention and functional assessment. Acad Emerg Med.

[2] Aminzadeh F, Dalziel WB (2002). Older adults in the emergency department: a systematic review of patterns of use, adverse outcomes, and effectiveness of interventions. Ann Emerg Med.

[3] Hughes JM, Freiermuth CE, Shepherd-Banigan M, Ragsdale L, Eucker SA, Goldstein K (2019). Emergency department interventions for older adults: a systematic review. J Am Geriatr Soc.

[4] Beauchet O, Fung S, Launay CP, Cooper-Brown LA, Afilalo J, Herbert P (2019). Screening for older inpatients at risk for long length of stay: which clinical tool to use?. BMC Geriatr.

[5] García-Pérez L, Linertova R, Lorenzo-Riera A, Vazquez-Diaz JR, Duque-Gonzalez B, Sarria-Santamera A (2011). Risk factors for hospital readmissions in elderly patients: a systematic review. QJM Int J Med.

[6] Reed RL, Isherwood L, Ben-Tovim D (2015). Why do older people with multi-morbidity experience unplanned hospital admissions from the community: a root cause analysis. BMC Health Serv Res.

[7] Wallace E, Stuart E, Vaughan N, Bennett K, Fahey T, Smith SM (2014). Risk prediction models to predict emergency hospital admission in community-dwelling adults. Med Care.

[8] Baldonado A, Hawk O, Ormiston T, Nelson D (2017). Transitional care management in the outpatient setting. BMJ Qual Improv Rep.

[9] Beauchet O, Launay CP, Fantino B, Lerolle N, Maunoury F, Annweiler C (2013). Screening for elderly patients admitted to the emergency department requiring specialized geriatric care. J Emerg Med.

[10] McCusker J, Dendukuri N, Tousignant P, Verdon J, Poulin de Courval L, Belzile E (2003). Rapid two-stage emergency department intervention for seniors: impact on continuity of care. Acad Emerg Med Off J Soc Acad Emerg Med.

[11] Ellis G, Marshall T, Ritchie C (2014). Comprehensive geriatric assessment in the emergency department. Clin Interv Aging.

[12] Launay C, de Decker L, Hureaux-Huynh R, Annweiler C, Beauchet O (2012). Mobile geriatric team and length of hospital stay among older inpatients: a case-control pilot study. J Am Geriatr Soc.

[13] Launay C, Annweiler C, de Decker L, Kabeshova A, Beauchet O (2013). Early hospital discharge of older adults admitted to the emergency department: effect of different types of recommendations made by a mobile geriatric team. J Am Geriatr Soc.

[14] Launay CP, Rivière H, Kabeshova A, Beauchet O (2015). Predicting prolonged length of hospital stay in older emergency department users: use of a novel analysis method, the artificial neural network. Eur J Intern Med.

[15] Preston L, Chambers D, Campbell F, Cantrell A, Turner J, Goyder E (2018). What evidence is there for the identification and management of frail older people in the emergency department? A systematic mapping review.

[16] Raîche M, Hébert R, Dubois MF (2008). PRISMA-7: a case-finding tool to identify older adults with moderate to severe disabilities. Arch Gerontol Geriatr.

[17] Hébert R, Raîche M, Dubois MF, Gueye NR, Dubuc N, Tousignant M (2010). Impact of PRISMA, a coordination-type integrated service delivery system for frail older people in Quebec (Canada): a quasi-experimental study. J Gerontol B Psychol Sci Soc Sci.

[18] Klijs B, Nusselder WJ, Looman CW, Mackenbach JP (2011). Contribution of chronic disease to the burden of disability. PLoS One.

[19] Beauchet O, Chabot J, Fung S, Launay CP (2018). Update of the 6-item brief geriatric assessment screening tool of older inpatients at risk for long length of hospital stay: results from a prospective and observational cohort study. J Am Med Dir Assoc.

[20] Bullard MJ, Musgrave E, Warren D, Unger B, Skeldon T, Grierson R (2017). Revisions to the Canadian emergency department triage and acuity scale (CTAS) guidelines 2016. CJEM..

[21] O’Caoimh R, Costello M, Small C, Spooner L, Flannery A, O’Reilly L (2019). Comparison of frailty screening instruments in the emergency department. Int J Environ Res Public Health.

[22] Fried LP, Tangen CM, Walston J (2001). Frailty in older adults : evidence for a phenotype. J Gerontol A Biol Sci Med Sci.

[23] Fried LP, Ferrucci L, Darer J, Williamson JD, Anderson G (2004). Untangling the concepts of disability, frailty and comorbidity: implications for improved targeting and care. J Gerontol A Biol Sci Med Sci.

[24] Mukamel DB, Chou CC, Zimmer JG, Rothenberg BM (1997). The effect of accurate patient screening on the cost-effectiveness of case management programs. Gerontologist..

[25] Moons P, De Ridder K, Geyskens K, Sabbe M, Braes T, Flamaing J (2007). Screening for risk of readmission of patients aged 65 years and above after discharge from the emergency department: predictive value of four instruments. Eur J Emerg Med.

[26] Manuel DG, Rosella LC, Hennessy D, Sanmartin C, Wilson K (2012). Predictive risk algorithms in a population setting: an overview. J Epidemiol Community Health.

